# T Cell Peptides Derived from Invasive Stages of *Schistosoma mansoni* as Potential Schistosomiasis Vaccine

**DOI:** 10.3390/jcm10030445

**Published:** 2021-01-24

**Authors:** Julio López-Abán, Belén Vicente, Elías Kabbas-Piñango, Juan Hernández-Goenaga, Javier Sánchez-Montejo, María Aguiriano, Esther del Olmo, Magnolia Vanegas, Manuel Alfonso Patarroyo, Antonio Muro

**Affiliations:** 1 Infectious and Tropical Diseases Group (e-INTRO), IBSAL-CIETUS (Biomedical Research Institute of Salamanca-Research Centre for Tropical Diseases at the University of Salamanca), Faculty of Pharmacy, University of Salamanca, Ldo Méndez Nieto s/n, 37007 Salamanca, Spain; belvi25@usal.es (B.V.); elias.kabbas@gmail.com (E.K.-P.); juhego@usal.es (J.H.-G.); S.Montejo@usal.es (J.S.-M.); maguiriano@usal.es (M.A.); 2 Department of Pharmaceutical Chemistry, IBSAL-CIETUS, Faculty of Pharmacy, University of Salamanca, Ldo Méndez Nieto s/n, 37007 Salamanca, Spain; olmo@usal.es; 3 Molecular Biology and Immunology Department, Fundación Instituto de Inmunología de Colombia (FIDIC), Carrera 50#26-20, Bogotá DC 111321, Colombia; magyvan@gmail.com (M.V.); mapatarr.fidic@gmail.com (M.A.P.); 4 Microbiology Department, Faculty of Medicine, Universidad Nacional de Colombia, Carrera 45#26-85, Bogotá DC 111321, Colombia

**Keywords:** *Schistosoma mansoni*, helminth vaccines, transcriptome, immunomodulator AA0029, synthetic peptide, ADAD vaccination system

## Abstract

Schistosomiasis is a parasitic disease that affects 143 million people in endemic countries. This work analyzed overexpressed sequences from the cercaria phase to the early schistosomulum phase using bioinformatics tools to predict host interaction and selected proteins for predicting T cell epitopes. The final peptides were chemically synthesized, and their toxicity was evaluated in vitro. Peptides were formulated in the Adjuvant Adaptation (ADAD) vaccination system and injected into BALB/c mice that were challenged with *S. mansoni* cercariae to assess protection and immunogenicity. A total of 39 highly expressed *S.* *mansoni* proteins were identified as being of potential interest. Three T cell peptides predicted to bind MHC mouse and human class II were synthesized and formulated for vaccination. SmGSP and SmIKE reduced the number of eggs trapped in the liver by more than 50% in challenged BALB/c mice. The liver of mice vaccinated with either SmGSP or SmTNP had a significantly reduced affected liver surface. Transcriptome-based T cell peptides elicit partial protection and could be candidates for a multiantigen vaccine.

## 1. Introduction

Schistosomiasis is a parasitic disease caused by contact with fresh water contaminated with cercariae of trematodes from the genus *Schistosoma*. It has been estimated that 143 million people are infected in 78 countries, mostly in sub-Saharan Africa [1]; annual mortality involves over 8800 people [2] and 693,000 disability-adjusted life years (DALYs) [3]. Chemoprophylaxis is the most established prevention measure in endemic countries. The widest used strategy consists of the mass drug administration of praziquantel, especially in children. Despite success in reducing morbidity, this program only reaches 13% of the population needing treatment [4]. The mass use of praziquantel is safe and efficacious but does not prevent reinfection; endemic populations therefore suffer from continuous reinfections. There is also a rising concern regarding the potential development of resistance to treatment [5]. Developing new complementary measures in the field of schistosomiasis prevention and control, i.e., an effective vaccine, is of paramount importance. Many antigens have been studied, but only a few have shown certain levels of protection against the disease. Only rSh28GST [6] and Sm14 [7] have reached phase II clinical trials and rSh28GST progressed to phase III clinical trials, though sufficient efficacy was not achieved [5].

Reverse vaccinology is a strategy involving rational antigen selection and vaccine development based on the information obtained from studied organisms’ immunological response and genomic sequences [8,9]. One strategy for finding target molecules consists of designing synthetic peptides from proteins expressed during critical stages of the parasite’s life cycle [10]. Bioinformatic tools enable selecting vaccine candidates by predicting peptides’ binding affinity for major histocompatibility complex (MHC) I or II [11]. MHC II can recognize extracellular antigens and can bind 13 to 18 amino acid-long peptides. T cells can trigger stronger immune responses after their antigen-presenting cell recognition, according to the peptides bound to MHC class II receptors [12]. Epitopes might be bioinformatically designed from parasite proteins, and their interaction with T or B cells might be predicted regarding vaccine design purposes [13].

The genomes of the three main schistosome species currently affecting humans have been described: *S. mansoni* [14], *S. japonicum* [15] and *S. haematobium* [16]. The *S. mansoni* genome has been sequenced [14], re-sequenced and partially annotated [17]. The development of next-generation sequencing (NGS) technologies and computer systems capable of managing and storing high-throughput analysis data has contributed to selecting new target vaccine candidate molecules [18,19,20]. A transcriptome comparing the cercaria, schistosomulum and adult stages is available for research [18]. Inhibition of parasite entry could reduce invasion through the skin, preventing disease transmission. In fact, differentially expressed sequences observed from the cercarial stage to the early schistosomulum stage therefore represent an interesting vaccination target.

This study identifies new vaccine candidates using the transcriptome for selecting highly expressed trans-stage antigens that could be involved in *S. mansoni* infective phase passage through the skin or the migratory phase. Three T cell epitopes able to bind MHC II were selected, synthesized and formulated using the adjuvant adaptation (ADAD) vaccination system. We evaluated the protection triggered by our new vaccine candidates in the terms of worm burden, eggs in tissues, liver damage and antibody response by immunizing BALB/c mice and a challenge involving *S. mansoni* cercariae.

## 2. Material and Methods

### 2.1. Mice and Parasites

Specific pathogen-free (SPF) 7-week-old female BALB/c mice (Charles River, Lyon, France) weighing 19–21 g were used. The animals were kept in standard conditions in the University of Salamanca’s Animal Experimentation facilities. Current Spanish and European Union regulations regarding animal experimentation were followed (L32/2007, L6/2013, RD53/2013 and Di2010/63/CE). Health and welfare statuses were monitored during the experiments according to FELASA guidelines. Mice were weighed and monitored during the experiments for signs of pain, anaphylactic shock, erythema at the injection site and changes in behavior. All efforts were made to minimize animal suffering. This study was approved by the University of Salamanca’s Bioethics Committee (registration CBE-335. *S. mansoni* LE was the parasite strain used in all experiments in our laboratory. Freshwater snails from the species *Biomphalaria glabrata* were used as intermediate host; these were infected when they were 4–8 mm in diameter with 7 miracidia of the parasite. They were kept in a 12/12 h light/dark cycle at 27 °C room temperature. Cercariae emission was induced after 30 days with light at 26 °C for 2 h. Counts were made in triplicate to obtain a dose of 150 cercariae in 0.7–1.2 mL of water to infect CD1 mice percutaneously.

### 2.2. Exploring the Schistosoma mansoni Transcriptome to Identify Potential Sources of Vaccine Candidates and Design T Cell Peptide Candidates

Overexpressed sequences in *S. mansoni* cercariae and early schistosomulum phases were selected by transcriptome sequencing [18]; a set of bioinformatics tools was used to assess their biological properties. The SignalP 4.1 Server (Center for Biological Sequence Analysis, Technical University of Denmark CBS-DTU, Lyngby, Denmark) was used for studying signal peptides in amino acid sequences [21]. The sequences were later analyzed using the WoLF PSORT protein subcellular location prediction tool (Computational Biology Research Centre, AIST, Tokyo, Japan) which uses several features, such as functional motifs, amino acid composition and sorting signals, to predict the subcellular location [22]. Sequences that contained signal peptides and were classified as extracellular were selected for further study. Putative CD4 T cell epitopes were predicted from the full-length sequence of the selected proteins using the SYFPEITHI v.1.0 tool (University of Tübingen, Tübingen, Germany) [23,24,25]. Haplotype H2-Ed from murine model BALB/c and six HLA-II human alleles (DRB1 0101, DRB1 0301, DRB1 0401, DRB1 0701, DRB11101 and DRB1 1501) were used for prediction. All epitopes of 15 amino acids and scoring over 20 in the murine haplotype and at least three different human haplotypes were selected.

### 2.3. Chemical Synthesis of T Cell Vaccine Candidates and Toxicity Study

Fifteen amino acid-long oligopeptides were chemically synthesized by solid-phase peptide synthesis at Fundación Instituto de Inmunología de Colombia (FIDIC), as described by Merrifield [26] and Houghten [27], using the t-Boc strategy and a benzhydrylamine (BHA) resin (0.7 mEq/mg). One cysteine and one glycine residue were added at both the amino and carboxyl-terminal to enable their polymerization via oxidization. Peptides were purified by reverse-phase high-performance liquid chromatography (RO-HPLC), characterized by MALDI-TOF mass spectrometry and lyophilized. Freeze-dried synthetic peptides were suspended in phosphate buffered saline solution (PBS) and concentrations were determined by a bicinchoninic acid (BCA) kit (Pierce, Rockford, IL, USA) prior to formulation in the ADAD system. *In vitro* toxicity was determined in J774.2 mouse macrophage cell cultures, measuring cell viability using the CytoTox96 Non-Radiactive Citotoxicity Assay (Promega), by following the manufacturer’s instructions [28]. Adherent cells were incubated for 72 h with each peptide in a range of 1–100 µL at 37 °C in 5% CO_2_ and results were compared to non-treated cells in percentages.

### 2.4. Vaccination with the ADAD System, Challenge and Necropsy

The immunization schedule was designed using the ADAD vaccination system. Briefly, the immunomodulator and adjuvant were formulated without an antigenic component and injected five days before immunization, allowing immune system adaptation to other vaccine components before using the antigen. Each injection consisted of a 30/70 water/oil ratio. Micelle was made with Montanide ISA763A GV non-mineral oil (SEPPIC, Paris, France) and an aqueous phase containing the synthetic immunomodulator AA0029 and the nonhemolytic saponins from *Quillaja saponaria* (Qs, Sigma, St. Louis, MO, USA) plus the antigen [29,30].

Female BALB/c mice were randomly allocated to five groups of six animals each [31,32]: the uninfected control group (Uninfected), those treated with the ADAD vaccination system using the AA0029 immunomodulator and Qs adjuvant and infected group (Adjuvant), those vaccinated with the SmGSP peptide formulated in ADAD with AA0029 and Qs and infected group (SmGSP), those vaccinated with the SmTNP peptide formulated in ADAD with AA0029 and Qs and infected group (SmTNP) and those vaccinated with the SmIKE peptide formulated in ADAD with AA0029 and Qs and infected group (SmIKE). Individual doses containing 10 µg of each peptide, 100 μg of AA0029 and 20 μg of Qs were emulsified by a handheld sonicator with the non-mineral oil at 100 μL final volume. Vaccination and two boosters were performed at two-week intervals via subcutaneous injection. Challenge with 150 ± 9 *S. mansoni* cercariae was performed percutaneously two weeks after completing the vaccination schedule. Mice were restrained with a mixture of ketamine (50 mg/kg, Imalgene1000, Merial, Lyon, France), diazepam (5 mg/kg, Valium10, Roche Farma SA, Madrid, Spain) and atropine (1 mg/kg, B. Braun, Madrid, Spain) by intraperitoneal injection. They were placed in the supine position; a plastic ring was placed on the abdomen with the help of adhesive tape, and a water suspension containing cercariae was applied. After 45 min, rings and the remaining water were removed. A lethal dose of 100 mg/kg of pentobarbital (Sigma) plus 50 IU/mL heparin was administered eight weeks after infection. All adult worms inside portal and mesenteric veins were recovered after perfusion with PBS plus 2 IU/mL heparin (Hospira Invicta SA, Alcobendas, Spain) through the left ventricle. The number of worms recovered was recorded considering couples, single males and single females. A section of the intestine and another piece of the liver from each mouse were also weighted and digested in 5% potassium hydroxide for 24 h at 37 °C to determine egg load. Digested samples were spun at 1200 rpm for 5 min and 5 mL concentrated egg suspension was counted using a McMaster chamber. Each sample was counted in triplicate and the number of eggs per gram (EPG) in intestine or liver was estimated. Three micrographs from each liver were collected using an Olympus camera adapted to a microscope (SZX9, Tokyo, Japan) to assess liver injury and Adobe Photoshop CS5 and ImageJ 1.45 software packages were used for calculating the affected surface of the liver. Blood samples were also collected at the beginning of the experiment (day 0 of the experiment), before the infection was induced (day 42) and before the necropsies of the animals at the end of the experiment (day 98).

### 2.5. Serum Antibodies Induced by Vaccination and Infection

Indirect ELISA measuring total IgG levels against the target peptides was performed to ascertain the presence of antibodies in sera samples following vaccination. Briefly, polystyrene 96-well plates (Costar 3369, Corning Inc, Glendale, USA) were coated with 1 μg of each synthetic peptide (SmGSP, SmTNP, SmIKE) per well in 100 μL carbonate buffer at pH 9.6 for 18 h at 4 °C. Three washes were then performed with 200 μL PBS/well, pH 7.2 and 0.05% Tween-20 (Sigma) (PBS-T); 2% BSA (Sigma) in PBS-T was then added and the plate was incubated at 37 °C for 1 h to avoid non-specific binding. Three washes were then made as described above. The serum sample from each mouse was incubated (1 h, 37 °C, 1:100 in PBS-T) and then washed as above. Peroxidase-coupled anti-mouse IgG from sheep (A5906, Sigma) was added at 1:1000 dilution in PBS-T, incubated again for 1 h at 37 °C and washed three times. O-phenylenediamine dihydrochloride (OPD) and H_2_O_2_ in citrate buffer pH 5 were added for the colorimetric reaction with the peroxidase. The reaction was stopped with 3N H_2_SO_4_ 50 μL/well and absorbance was measured at 492 nm using a Multiskan GO1510 spectrophotometer (Thermo Fischer Scientific, Vantaa, Finland).

Soluble adult *S. mansoni* somatic antigen (SoSmAWA) was used to quantify the specific IgG, IgG1 and IgG2a to measure the antibody-mediated immune response against infection [33]. Briefly, 96-well plates were covered with 0.5 μg SoSmAWA per well. Infected mouse sera were diluted at 1:100 and anti-IgG, anti-IgG1 and IgG2a secondary antibodies at 1:1000. SoSmAWA was obtained from 10 *S. mansoni* couples/mL in PBS with a commercial protease inhibitor (Complete Mini EDTA-Free, Roche 04 693 159 001) to avoid antigen degradation. The mixture was homogenized, frozen and thawed, sonicated and then centrifuged at 30,000 *g* for 30 min at 4 °C. Supernatant protein concentration was determined using a Micro BCA™ Protein Assay Kit (Thermo Scientific Waltham, MA, USA), following the manufacturer’s instructions.

### 2.6. Statistical Analysis

The results were expressed as the mean and standard error of the mean (SEM). The percentage of reduction (R) regarding worm burden, eggs in tissues and affected liver surface was calculated as follows: R = (C − T/C) × 100, where C is the mean for the adjuvant control group and T is the mean for the treated groups. The Simfit V7.3.7 statistical package was used for further processing data and their statistical analysis. First, a Bartlett test was performed to check data homoscedasticity. Once variance homogeneity had been verified, ANOVA analysis was carried out followed by post hoc Tukey’s honest significance test (HSD) to determine significant differences between study groups. All *p*-values under 0.05 were considered significant. Charts were designed with R software using the packages “ggplot2” and “ggpubr”.

## 3. Results

### 3.1. In Silico Identification of Potential Vaccine Candidates

**Figure 1:**
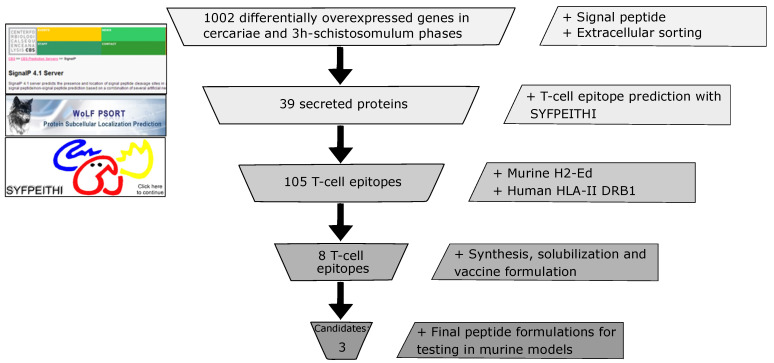
T cell epitopes prediction and selection derived from overexpression in *Schistosoma mansoni* from the cercaria stage to 3-h schistosomulum stage having highly potential host–parasite interaction based on the presence of a signal peptide (SignalP 4.1), subcellular location (WoLF PSORT) and T cell peptide prediction (SYFPEITHI).

The selection of new vaccine candidates derived from the transcriptome was based on the results of a study comparing protein expression between different of *S. mansoni* stages using a high-throughput RNAseq technique [18]. Our search strategy can be summarized as the prediction of functional motifs and subcellular location and the prediction of potential T cell epitopes and their interaction with the known MHC II (Figure 1). We retrieved the sequences of 1002 genes up-regulated in the cercaria and 3-h schistosomulum phases that could be involved in the infection process. Analyzing the sequences for the presence of a signal peptide (SignalP 4.1) and other protein features such as amino acid composition, functional motifs or sorting signals (WoLF PSORT) gave 39 *S. mansoni* proteins having a signal peptide which were marked as extracellular. The SYFPEITHI tool v.1.0 was used for predicting T cell epitope antigens having high binding affinity for MHC class II from mice and six human alleles; 105 peptides from 28 proteins scored more than 20 using the model. The predicted epitopes were ranked according to their affinities for mouse and human alleles. Peptides potentially binding to H2-Ed in BALB/c mice and to at least three out of the six HLA-DRB1 human alleles were selected, giving eight T cell epitopes in eight different proteins.

### 3.2. T Cell Epitope Characterization, Synthesis and Toxicity Assessment

Five of the eight initially predicted T cell epitopes were discarded due to experimental difficulties regarding synthesis, solubilization and vaccine formulation. The three remaining peptides’ biological function was predicted, and cytotoxicity was assessed (Table 1). Peptide GSPYLWIPSKKCDPS (SmGSP) came from the GeneDB sequence Smp_136730, a subfamily A1A unassigned peptidase (A1 pepsin family). The TNPYHLIYPQKSPAL peptide (SmTNP) was predicted from the Smp_136920 sequence, a polypeptide having putative N-acetyl-galactosaminyl transferase activity involved in the binding of calcium and magnesium ions. The third peptide, IKELEDKYNLRLYSA (SmIKE), was predicted from the Smp_021460 sequence, annotated as a protein having putative bacteria glutamine synthetase activity involved in glutamine biosynthesis and nitrogen metabolism. No toxicity was found, given that over 95% of the J774.2 macrophages were viable after three days of exposure to each of the peptides at 1–100 µg/mL.

### 3.3. Antibody Response to Vaccination with SmGSP, SmTNP and SmIKE

**Figure 2:**
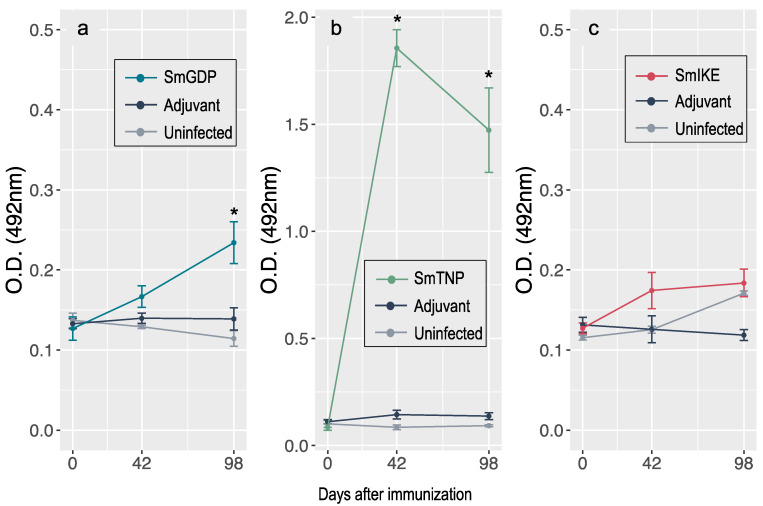
Specific IgG levels (means ± SEM) by indirect ELISA elicited by (**a**) SmGSP peptide, (**b**) SmTNP peptide and (**c**) SmIKE peptide formulated in the adjuvant adaptation (ADAD) vaccination system, with AA0029 and *Quillaja saponaria* saponins, 0, 42 and 98 days after the first immunization in BALB/c mice. * Statistically significant differences compared to the uninfected group (*p* < 0.05).

The presence of specific antibodies produced against a given vaccine enables monitoring the level of immunization achieved after vaccination. The group vaccinated with SmTNP had greater IgG anti-SmTNP production compared to the uninfected control group at six weeks after the first vaccination (F_(2,15)_ = 196.7 *p* < 0.001 HSD *p* < 0.001) and the levels remained high until the end of the experiment (F_(2,15)_ = 22.7 *p* = 0.002 HSD *p* = 0.001) (Figure 2b). Mice vaccinated with SmGSP also had statistically significantly high IgG levels 96 days after the first immunization (F_(2,15)_ = 11.4 *p* = 0.015 HSD *p* = 0.014) (Figure 2a). Animals vaccinated with SmIKE had no increase in antigen-specific IgG (Figure 2c).

### 3.4. Reduction in Worm Recovery and Egg Burden

Data obtained by parasitological techniques led to estimating our vaccine candidates’ protective capacity. Adult worm burden and eggs trapped in the liver and gut were studied in mice vaccinated with or without our peptides. Mice vaccinated with SmGSP or SmTNP had slight reductions regarding total number of worms recovered (19% and 36%) compared to the adjuvant control group; however, the differences found were not statistically significant (Table 2). Mice vaccinated with SmIKE had no change concerning the observed variables (Table 2). Vaccination with all three candidates did not significantly modify the male/female adult worm ratio.

**Figure 3:**
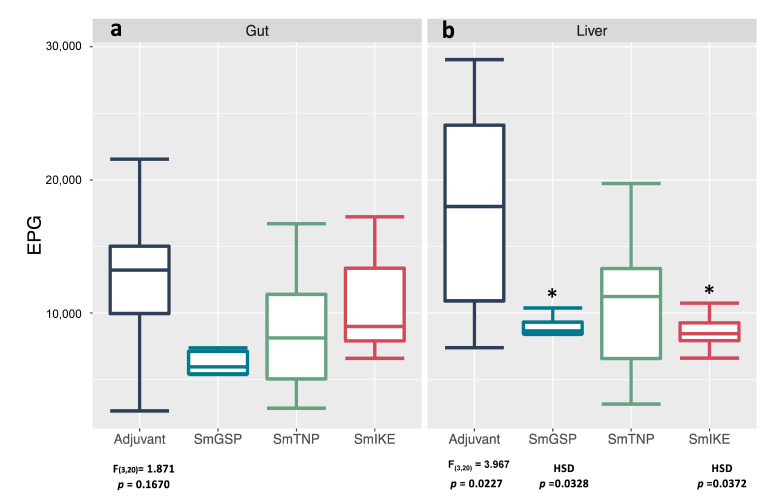
Egg burden in tissues (eggs per gram, EPG): (**a**) in the gut and (**b**) liver of BALB/c mice vaccinated with SmGSP, SmTNP and SmIKE formulated in the ADAD vaccination system with AA0029 and *Quilaja saponaria* saponins at eight weeks post-*Schistosoma mansoni* challenge. * Statistically significant differences (*p* < 0.05) were determined by analysis of variance (ANOVA) and post hoc Tukey’s test (HSD).

The number of eggs trapped in tissue is one of the main indicators of schistosomiasis severity. Mice vaccinated with SmGSP showed a remarkable 53% reduction regarding liver EPG (*p* = 0.0328) and a 50% reduction regarding small intestine EPG compared to the adjuvant control group. The group vaccinated with SmTNP had non-significant reductions in EPG in the liver (40%) and gut (30%) compared to the control group. Animals vaccinated with SmIKE had a 52% reduction in EPG in the liver (*p* = 0.0372) but a non-significant reduction in the gut (15%) compared to the adjuvant control group (Figure 3). A reduction in egg production per female would be a desirable feature of an anti-schistosomiasis vaccine as it would help reduce egg passing. Small reductions in fecundity were observed (15–26%), calculated as eggs in the liver or gut per female, in almost all vaccinated groups; however, none of the reductions were statistically significant.

### 3.5. Protection against Liver Injury

**Figure 4:**
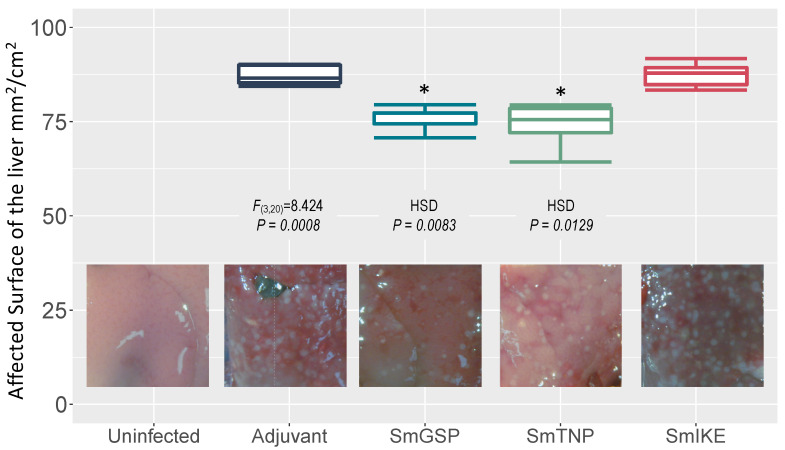
Affected surface of the liver (mm^2^/cm^2^) in BALB/c mice vaccinated with SmGSP, SmTNP and SmIKE formulated in the ADAD vaccination system with AA0029 and *Quillaja saponaria* saponins at eight weeks post-*S. mansoni* challenge. * Statistically significant differences (*p* < 0.05) were determined with analysis of variance (ANOVA) and post hoc Tukey’s test (HSD).

Hepatic lesions serve as an indicator of immune system reaction intensity and the ability of schistosome eggs to induce damage. A significant reduction in the affected liver surface 8 weeks post-challenge was observed in mice vaccinated with SmGSP (75.9 ± 1.2, 13.1% reduction, *p* = 0.0083) and SmTNP (76.5 ± 3.9, 12.4% reduction, *p* = 0.0129) compared to the adjuvant control group (87.3 ± 1.0) (Figure 4). The group vaccinated with SmIKE (87.4 ± 1.2) did not show any reduction regarding liver damage.

### 3.6. Immune Response against the Infection

Specific IgG, IgG1 and IgG2a against SoSmAWA responses were studied using indirect ELISA. All infected groups had higher total IgG levels compared to the uninfected group. All infected groups had high and significant IgG1 production at 8 weeks post-challenge compared to the uninfected group, except for the group vaccinated with SmGSP that had a non-significant increase. There were non-significant differences regarding IgG and IgG1 levels between groups vaccinated with SmGSP, SmTNP and SmIKE. No differential IgG2a antibody production against SoSmAWA was observed in any of the groups throughout the experiment (Table 3).

## 4. Discussion

Mass drug administration of praziquantel is still the standard drug used for controlling and eliminating schistosomiasis. Therapeutic failures may occur, reinfections are not avoided and there is the risk of developing resistance to praziquantel [4]. The search for alternative drugs to praziquantel [34,35] and effective vaccines [36] is thus still necessary along with improving access to water, sanitation or eliminating intermediate hosts. New vaccine candidates are needed to provide better protection than that induced by classic candidates, such as rSh28GST, Sm-TSP-2 and Sm14 [5]. These candidates have been seen in extracellular vesicles in the schistosomulum and adults in non-conventional secretion different to signal peptides [37,38]. Transcriptomes are interesting sources of new vaccine candidates because they provide information regarding the expression of proteins and metabolic pathway activity for almost all phases of schistosomes’ life cycles and could provide as-yet-unknown candidates [18,39,40]. Our study identified 1002 overexpressed sequences during development from the cercaria to the early schistosomulum phases in the transcriptome study of *S. mansoni* [18]. We considered that high expression regarding early schistosomula adaptation to a host could provide new molecules apt for participating in invasion. We also believed that the secreted proteins could interact more easily with a particular host and play a role in skin invasion or migration to the lungs. Consequently, we identified 39 proteins when searching for proteins having signal peptides.

One strategy for predicting new vaccine candidates consists of identifying small peptides capable of stimulating MCH class II and inducing a protective T cell-mediated response [41]. Peptides could be considered an alternative to using whole proteins. Subunit vaccines against infectious diseases could represent a more feasible and accurate option because they are easier to manufacture and store than conventional antigens [42]. Our strategy was to predict new high-affinity, short-chain peptides potentially recognizable by mouse H2-Ed and human MHC-II alleles for eliciting initial immune protection in a BALB/c murine model for schistosomiasis. We obtained up to 105 15-mer peptides from selected proteins; however, only three were selected after scoring them and comparing them to T cell peptides fitting H2-Ed and three human MHC-II. The three peptides were obtained from three proteins which had not been used as vaccine candidates in *S. mansoni* beforehand. The SmGSP 15 amino acid-long T cell peptide was obtained from the Smp_136730 sequence, a peptidase from the A1 subfamily. Peptidases have been widely used for designing linear and conformational cathepsin-derived epitopes [43,44,45]. SmTNP belongs to an N-acetyl galactosaminyl transferase involved in o-glycan biosynthesis using calcium and magnesium ions as cofactors. This activity has been described in *S. mansoni* excretory–secretory products involved in interfering with various internal defense functions [46]. SmIKE was designed on a sequence having homology with a bacterial glutamine synthetase involved in nitrogen provision for producing purines, pyrimidines and amino acids by glutamine synthesis. A glutamine synthetase has been described having high expression in the *S. japonicum* 21-day schistosomulum and adult worms of that and has been suggested as a drug target [47]. None of these three proteins have been identified in extracellular vesicles studies in our work [48].

We next formulated our candidates in the adjuvant adaptation (ADAD) vaccination system using non-hemolytic *Quillaja saponaria* saponins and a synthetic immunomodulator (AA0029) in an emulsion with Montanide non-mineral oil to obtain a feasible long-term delivery system for use in developing a vaccine against schistosomiasis [33]. All SmGSP, SmTNP and SmIKE T cell epitopes induced partially significant protection in BALB/c mice with experimental schistosomiasis in terms of eggs per gram of liver and hepatic damage. Slight reductions in worm recovery and eggs trapped in the gut were also observed. These results are comparable to those obtained using fractions of other candidates, such as Kunitz domain-containing proteins [49,50], but protection was low compared to other T cell epitopes [51] or FABP, GST and TSP-2 [36]. The sample size was a limitation concerning our results, considering natural variation in worm recovery, egg count and liver lesions. Alternative more powerful delivery systems could improve vaccination results [52]. The candidates’ immunogenicity was monitored by ELISA and it was observed that SmTNP triggered high specific IgG antibody levels in vaccinated, non-infected mice. Moreover, mice vaccinated with SmGSP also induced a specific immune response later. However, SmIKE-vaccinated mice did not elicit a high antibody response. In fact, mice vaccinated with SmTNP or SmGSP and after challenged had better protection data; this agrees with the role attributed to IgG antibodies regarding protection against schistosomiasis [53]. SmIKE did not trigger a specific antibody response during the experiment; this was a well-known possibility as using short peptides entails the difficulty of foreseeing their immunological properties in silico [54]. Our group has observed that B cell peptides or the whole original protein elicited more antibodies than T cell peptides [13,51]. Infections also induced high IgG and IgG1 levels against crude extracts such as SoSmAWA, while there was no production against IgG2a, as has appeared in other experimental studies in mice [33]. Studying secreted proteins in extracellular vesicles could complete the repertoire of potentially interesting functional proteins for vaccination [38]. Moderate protection induced by a vaccine in humans could be sufficient to reduce the associated mortality and morbidity [55].

These results represent a starting point for the rational design of new vaccine candidates based on T cell epitopes derived from transcriptome analysis of *S. mansoni* invasive stages. The SmGSP, SmTNP and SmIKE T cell peptides predicted in proteins expressed in host skin during *S. mansoni* penetration and early migratory phases conferred moderate protection in terms of eggs trapped in the liver and hepatic lesions in a murine model. These findings support the importance of transcriptome studies for finding new vaccine candidates. Completing a broad repertoire with synergic activity would be a milestone on the path to ensuring an effective vaccine against schistosomiasis.

## Figures and Tables

**Table 1:** Characterization of the peptides selected as vaccine candidates from highly expressed proteins in cercaria to 3-h schistosomulum with highly potential host–parasite interaction. Signal peptides were predicted by SignalP 4.1 and T cell peptides fitting human and mouse alleles by SYFPEITHI. Peptides’ potential cytotoxicity was measured as the percentage of J774.2 macrophage viability by the third day of culture.

	SmGSP	SmTNP	SmIKE
GeneDB	Smp_136730	Smp_136920	Smp_021460
Genebank	CCD78846	CCD79787	CAZ29822
Protein length	401	447	555
Product	Subfamily A1A unassigned peptidase	Polypeptide having N-acetyl galactosaminyl transferase activity	Putative bacteria glutamine synthetase
Signal peptide position	1–17	1–23	1–23
T epitope	GSPYLWIPSKKCDPS	TNPYHLIYPQKSPAL	IKELEDKYNLRLYSA
Position	97–111	271–285	84–98
Fitting murine MHC (Score < 20)	H2-Ed	H2-Ed	H2-Ed
Fitting human MHC (Score < 20)	DRB1*0101 DRB1*0401 DRB1*0701	DRB1*0101 DRB1*0401 DRB1*1101	DRB1*0301 DRB1*0701 DRB1*1501
J774.2 macrophage viability (%)	96.8	97.9	96.4

**Table 2:** Recovery and reduction percentage (R) of total worms, females and males mean and standard error of mean (SEM) in BALB/c mice vaccinated with SmGSP, SmTNP and SmIKE formulated in the ADAD vaccination system, with AA0029 and *Quillaja saponaria* saponins at eight weeks post-*S. mansoni* challenge. Statistically significant differences (*p* < 0.05) were determined by analysis of variance (ANOVA).

Group	Total Worms	Mean ± SEM	R (%)	Female Worms	Mean ± SEM	R (%)	Male Worms	Mean ± SEM	R (%)
**Adjuvant**	26, 27, 25, 35, 18, 18	24.8 ± 2.6	-	18, 18, 16, 19, 13, 10	15.7 ± 1.4	-	8, 9, 9, 16, 5, 8	9.2 ± 1.5	-
**SmGSP**	6, 32, 16, 8, 21, 13	16.0 ± 3.9	36	2, 21, 9, 4, 12, 7	9.2 ± 2.8	42	4, 11, 7, 4, 9, 6	6.8 ± 1.1	26
**SmTNP**	20, 7, 21, 14, 22, 37	21.2 ± 4.1	19	10, 4, 11, 10, 13, 24	12.0 ± 2.7	23	10, 3, 10, 4, 9, 13	8.2 ± 1.6	11
**SmIKE**	29, 14, 23, 27, 37, 39	28.2 ± 3.8	-	17, 7, 14, 14, 27, 24	17.2 ± 3.0	-	12, 7, 9, 13, 10, 15	11.0 ± 1.2	-
**ANOVA**		F_(3,20)_ = 2.153 *p* = 0.125			F_(3,20)_ = 2.016 *p* = 0.144			F_(3,20)_ = 1.662 *p* = 0.207	

**Table 3:** Specific IgG levels by indirect ELISA against SoSmAWA antigen in BALB/c mice vaccinated with SmGSP, SmTNP and SmIKE formulated in the ADAD vaccination system with immunomodulator AA0029 at eight weeks post-*S. mansoni* challenge. * Statistically significant differences (*p* < 0.05) were determined by analysis of variance (ANOVA) and post hoc Tukey’s test (HSD).

Group	IgG	IgG1	IgG2a
**Uninfected**	0.194 ± 0.002	0.201 ± 0.004	0.111 ± 0.007
**Adjuvant**	0.577 ± 0.101 * HSD *p* = 0.002	0.653 ± 0.010 * HSD *p* = 0.003	0.138 ± 0.016
**SmGSP**	0.634 ± 0.049 * HSD *p* < 0.001	0.522 ± 0.050	0.210 ± 0.065
**SmTNP**	0.599 ± 0.047 * HSD *p* < 0.001	0.719 ± 0.094 * HSD *p* < 0.001	0.126 ± 0.012
**SmIKE**	0.697 ± 0.061 * HSD *p* < 0.001	0.703 ± 0.104 * HSD *p* = 0.001	0.153 ± 0.022
**ANOVA**	F_(4,25)_ = 10.60 *p* < 0.001	F_(4,25)_ = 7.62 *p* < 0.001	F_(4,25)_ = 1.43 *p* = 0.2543

## Data Availability

Data are available on request from the corresponding author.
